# Hard-Shelled Glycol Chitosan Nanoparticles for Dual MRI/US Detection of Drug Delivery/Release: A Proof-of-Concept Study

**DOI:** 10.3390/nano13152227

**Published:** 2023-08-01

**Authors:** Simona Baroni, Monica Argenziano, Francesca La Cava, Marco Soster, Francesca Garello, David Lembo, Roberta Cavalli, Enzo Terreno

**Affiliations:** 1 Molecular and Preclinical Imaging Centers, Department of Molecular Biotechnology and Health Sciences, University of Torino, Via Nizza 52, 10126 Torino, Italy; simona.baroni@unito.it (S.B.); psiedoll@gmail.com (F.L.C.); francesca.garello@unito.it (F.G.); 2 Department of Drug Science and Technology, University of Torino, Via P. Giuria 9, 10125 Torino, Italy; monica.argenziano@unito.it (M.A.); marco.soster@unito.it (M.S.); 3 Department of Clinical and Biological Sciences, University of Torino, S. Luigi Gonzaga Hospital, Regione Gonzole, 10, 10043 Orbassano, Italy; david.lembo@unito.it

**Keywords:** theranostics, MRI, ultrasound imaging, nanobubbles, Gd(III) complexes, relaxometry

## Abstract

This paper describes a novel nanoformulation for dual MRI/US in vivo monitoring of drug delivery/release. The nanosystem was made of a perfluoropentane core coated with phospholipids stabilized by glycol chitosan crosslinked with triphosphate ions, and it was co-loaded with the prodrug prednisolone phosphate (PLP) and the structurally similar MRI agent Gd-DTPAMA-CHOL. Importantly, the in vitro release of PLP and Gd-DTPAMA-CHOL from the nanocarrier showed similar profiles, validating the potential impact of the MRI agent as an imaging reporter for the drug release. On the other hand, the nanobubbles were also detectable by US imaging both in vitro and in vivo. Therefore, the temporal evolution of both MRI and US contrast after the administration of the proposed nanosystem could report on the delivery and the release kinetics of the transported drug in a given lesion.

## 1. Introduction

Theranostics is a promising strategy for precision medicine, where in vivo diagnostic imaging provides support and improves therapeutic treatment effectiveness [1,2]. This concept has gained great interest owing to the remarkable progress achieved in the field of nanomedicine [3,4]. Indeed, the use of nanoparticulate systems has the potential of providing multifunctional modalities with the versatility of loading and delivering a wide array of different materials.

The tight integration between pharmacology, advanced nanotechnology, and imaging probe chemistry may significantly improve the outcomes of therapeutic treatments, opening new avenues in the field of personalized medicine.

Many nanotheranostic systems have been developed with the goal of improving anticancer therapies [5,6], being able to affect the biodistribution of the transported drug. To make the nanosystems “visible” inside the body, allowing an in vivo assessment of the biodistribution, specific contrast agents need to be included in the nanoformulation, whose physicochemical properties depend on the imaging technology of choice (e.g., paramagnetic or superparamagnetic systems for magnetic resonance imaging (MRI), radiotracers for PET/SPECT, gas-filled systems for ultrasound (US) imaging).

The progress in the development of multimodal imaging protocols has made possible the successful combination of different imaging techniques in order to further expand their diagnostic a/nd theranostic potential. Recently, echogenic gas-filled microbubbles have been investigated for the purpose of enabling dual US/MRI detection [7,8,9,10,11,12]. Representative examples include theranostic lipid-based microbubbles loaded with a paramagnetic Gd(III) complex designed to guide focused ultrasound surgery [7], or albumin-shelled Gd-DTPA-loaded microbubbles to detect intracerebral hemorrhage [8].

Unlike microbubbles, their smaller analogs nanobubbles (NBs) are able to extravasate out of the vasculature, allowing their accumulation in the tumor stroma. Consequently, NBs have great potential to be used as innovative nanocarriers with improved biodistribution [13,14,15,16,17,18].

So far, the design of NBs for theranostic purposes has been mainly focused on US imaging, MRI, or dual US/MRI detectable systems, where the choice of the MRI agent to be loaded is primarily driven by the detection sensitivity of the probe in order to facilitate the visualization of the nanosystem’s delivery [19,20,21,22,23,24,25,26,27,28]. In this scenario, it is noteworthy that only a few systems have been reported where NBs were also loaded with a drug [25,26,27,28].

However, the visualization of the drug release, which is a very important process affecting the overall performance of the nanomedicine, needs to be investigated more deeply. For this purpose, the imaging probe should act as a drug surrogate, i.e., it should mimic the drug release properties. This aim can be achieved by choosing an imaging probe whose physicochemical properties are similar to those of the co-transported drug. Furthermore, the imaging signal generated by the probe has to show a modulation depending on whether it is transported by the nanosystem or released.

The aim of this work was to develop bimodal US/MRI NBs, where, in principle, echography can give information about the in vivo delivery and accumulation of the nanosystem at the pathological region, and MRI can provide an imaging response related to the release kinetics of the transported drug. 

Interestingly, it is worth noting that nanobubbles can be also used as sonosensitive drug delivery systems due to their ability to release the contents upon local exposure to ultrasound [29,30,31].

The herein purposely tuned NBs were composed of a hard shell consisting of crosslinked glycol chitosan filled with perfluoropentane (PFP). PFP has a boiling point of 29 °C; therefore, this formulation is referred to as nanobubbles for the sake of simplicity, but it would be more correct to use the term nanodroplets, with PFP being a liquid at room temperature. PFP can undergo a phase change to a gas upon insonation. 

**Figure 1:**
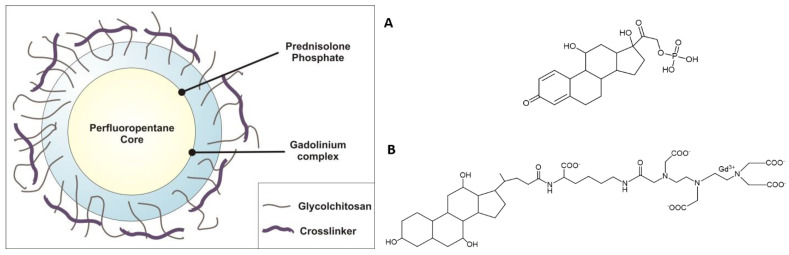
(**Left**): Schematic representation of the theranostic NBs developed in this work. (**Right**): Chemical formulae of prednisolone phosphate (**A**) and (**B**) the MRI agent Gd-DTPAMA-CHOL.

The NBs were loaded with both the steroid-like anti-inflammatory/antitumor prodrug prednisolone phosphate (PLP) and the structurally similar MRI contrast agent Gd-DTPAMA-CHOL (Figure 1).

This work illustrates the preparation and the characterization of the NBs, which includes the ability of the system to generate MRI and US contrast. Finally, the NBs’ ability to be visualized in contrast-enhanced US imaging was tested on a mouse model of prostate cancer.

## 2. Materials and Methods

### 2.1. Materials

The Gd-DTPAMA-CHOL complex [32] was kindly provided by Bracco Imaging S.p.A. (Colleretto Giacosa, TO, Italy). Ethanol 96° was purchased from Carlo Erba (Milan, Italy). DL-disteroyl-phosphatidylcholine (DSPC), perfluoropentane (PFP), glycol chitosan (GC), sodium triphosphate pentabasic (STP), prednisolone phosphate (PLP), and disteroyl-phosphatidylethanolamine (Lissamine-Rhodamine B) (Lipid-Rho) were purchased from Avanti Polar (Alabaster, AL, USA). Coumarin 6 was purchased from Acros Organics (Geel, Belgium). 

Gd-DTPAMA-CHOL complex 22 was kindly provided by Bracco Imaging S.p.A. (Colleretto Giacosa, TO, Italy). Ultrapure water was obtained using a 1–800 Milli-Q system (Millipore, Burlington, MA, USA). DMEM, FBS, glutamine, penicillin, and streptomycin were provided by Lonza (Verviers, Belgium).

### 2.2. Determination of PLP’s Partition Coefficient and Critical Micellar Concentration

To determine the partition coefficient of PLP between the PFP core of the nanodroplets and the aqueous phase, 1 mg of PLP was dissolved in 3 mL of filtered water and then mixed with 3 mL of PFP or PFP/DSPC mixture. The systems were shaken for 30 min at room temperature in the dark. The aqueous phase was then separated, diluted, and analyzed for the determination of PLP content, as described below. 

The critical micellar concentration (CMC) of PLP was determined by measuring the surface tension of PLP using the Du Noüy ring method with a Kruss tensiometer (Hamburg, Germany). The surface tension of a series of PLP solutions with increasing concentrations was measured. The CMC was obtained from the intersection between the straight regression line of the linearly dependent region and the straight line passing through the plateau.

### 2.3. Preparation of the Theranostic Glycol Chitosan Nanobubbles

A multistep method was purposely tuned to prepare the Gd-DTPAMA-CHOL and PLP co-loaded nanodroplets. Firstly, 300 microliters of an ethanolic solution of DSPC (3% *w*/*w*) was added under stirring to 350 microliters of perfluoropentane (PFP) at room temperature. Then, 4.8 mL of ultrapure water was slowly added to the mixture (under mild stirring) until the formation of an emulsion. Subsequently, the system was homogenized for two minutes at 12,000 rpm using a high-shear homogenizer (Ultraturrax, IKA, Germany) in an ice bath. After leaving the emulsion at 37 °C for 15 min (to allow for the homogenization of the suspension), an aqueous solution of glycol chitosan (pH = 5.0, 2.7% *w*/*w*) was added dropwise to form the nanobubbles’ polymeric shell. After 15 min under stirring, an aqueous solution of sodium triphosphate (0.1% *w*/*w*) was added dropwise. To obtain the theranostic nanoformulation, the Gd(III) complex at a concentration of 1 mM and PLP at a concentration of 4.5 mM were added to the ethanolic solution of DSPC. After the preparation, the unloaded Gd(III) complex and PLP (as well as other soluble components not entrapped in the nanosystem) were removed by dia-ultrafiltration using a TCF2 system (Amicon) with a dialysis membrane cutoff of 30 kDa. The thus-obtained theranostic nanodroplets were stored at 4 °C.

For the preparation of fluorescent nanobubbles, coumarin 6 (λex 443 nm, λem 505 nm) was dissolved directly in the PFP solution, while Lipid-Rho (λex 553 nm, λem 627 nm) was dissolved in the ethanolic solution of DSPC at a concentration of 0.1% *w*/*w*. Then, the preparation was carried out as described above for the theranostic nanobubbles.

### 2.4. Physical Characterization and Stability Assessment of the Nanobubbles

The mean hydrodynamic diameter, polydispersion index, and zeta potential values were determined by dynamic light scattering (DLS) using a 90 Plus instrument (Brookhaven, NY, USA) at a scattering angle of 90° and a temperature of 25 °C. The nanodroplet samples were diluted with filtered water. Each measured value was the average of 10 readouts. For zeta potential determination, samples were placed in the electrophoretic cell, where an electric field of about 14 V/cm was applied. 

The morphology of the nanobubbles was evaluated by transmission electron microscopy (TEM) using a Philips CM10 instrument (Eindhoven, the Netherlands), and by scanning electron microscopy (SEM) using a Leica Stereoscan 410 (Wetzlar, Germany). For TEM measurements, a diluted aqueous suspension of nanobubbles was sprayed on formvar-coated copper grid and air-dried before observation. For SEM experiments, a drop of the diluted aqueous suspension was placed on a stub and subsequently metalized. Moreover, the nanobubbles were also observed using a Motic AE31 optical and fluorescence microscope (Motic, Barcelona, Spain).

The osmolarity of the theranostic nanobubbles was determined at 25 °C using a Knauer osmometer, whereas the viscosity was determined at 25 °C using a Ubbelohde capillary viscosimeter (Schott Gerate, Mainz, Germany). 

The physical stability of the nanoparticles was evaluated by measuring their size and morphology over time at 25 °C. The shell and core stability were evaluated using optical microscopy. The formulation’s stability was also monitored at 37 °C in rat blood. 

### 2.5. Determination of the Hemolytic Activity

For the determination of the hemolytic activity, 100 microliters of nanodroplets was incubated at 37 °C for 90 min with diluted rat blood (1:4 *v*/*v*) obtained by adding freshly prepared PBS at pH = 7.4. 

After incubation, the blood was centrifuged at 1000 rpm for 5 min to separate plasma. The amount of hemoglobin released due to hemolysis was determined spectrophotometrically (absorbance readout at 543 nm using a Du730 spectrophotometer, Beckman Coulter, Fullerton, CA, USA). The hemolytic activity was calculated using nanodroplet-free diluted blood as a reference. Complete hemolysis was induced by the addition of ammonium sulfate (20% *w*/*v*).

In addition, optical microscopy was used to evaluate changes in red blood cell morphology after incubation with the theranostic nanosystem. 

### 2.6. Quantitative Determination of Loaded PLP

Loaded PLP was quantified spectrophotometrically (DU730 instrument, Beckman Coulter, Fullerton, CA, USA), measuring the absorbance at 243 nm after suitable dilution of the samples.

The PLP concentration was calculated from a calibration curve obtained starting from a stock solution containing a known amount of PLP in filtered water. The stock solution was then diluted with water to obtain a series of PLP solutions that were analyzed spectrophotometrically. A linear calibration curve was obtained over the concentration range of 0.5–20 μg/mL, with a regression coefficient of 0.998.

### 2.7. In Vitro Release Experiments 

The in vitro release kinetics of both PLP and Gd-DTPAMA-CHOL from the nanosystem was evaluated in a multi-compartment rotating cell with a cellulose membrane (cutoff = 12,000 Da) that kept the donor separated from the receiving compartment. The PLP content was determined spectrophotometrically, as described above. The Gd content was determined by ICP-MS (Spectro Genesis ICP-OES spectrometer, Spectro Analytical Instruments, Kleve, Germany) after sample mineralization under microwave heating at 160 °C for 40 min (Milestone MicroSYNTH Microwave lab station equipped with an optical fiber temperature control and HPR-1000/6M six-position high-pressure reactor, Bergamo, Italy). Moreover, PLP’s in vitro release kinetics was investigated after the application of US, using the dialysis bag technique. The dialysis bag, containing the donor phase was placed in 100 mL of the receiving phase and exposed to US for 5 min (US frequency 2.5 ± 0.1 MHz). The release of PLP from the nanodroplets/bubbles was determined up to 24 h post-insonation.

All of the in vitro release experiments were conducted in triplicate (n = 3).

To investigate the release kinetics of the drug from the theranostic nanodroplets/bubbles, mathematical models were tested (i.e., zero-order, first-order, Higuchi, and Korsmeyer–Peppas). From the release regression equation, the correlation coefficient R^2^ was calculated to evaluate the best-fitting profile.

### 2.8. Cell Viability and Cytotoxicity Assay

The cell viability and cytotoxicity of the blank and theranostic nanodroplets/bubbles were evaluated on three human cell lines: low-passage-number human embryonic lung fibroblasts (Helf), human epithelial adenocarcinoma (HeLa) cells, and human prostatic carcinoma cells (PC-3). Helf cells were grown as monolayers in minimum essential medium (MEM; Sigma, St. Louis, MO, USA) supplemented with 10% FBS, 1 mM sodium pyruvate (Gibco-BRL, Gaithersburg, MD, USA), and 1% antibiotics. HeLa cells were propagated in Dulbecco’s modified Eagle’s medium (DMEM; Sigma, St. Louis, MO, USA) supplemented with heat-inactivated 10% fetal bovine serum (FBS) (Gibco-BRL, Gaithersburg, MD, USA) and 1% antibiotic–antimycotic solution (Zell Shield, Minerva Biolabs GmbH, Berlin, Germany). Human prostatic carcinoma cells (PC-3) were cultured in Dulbecco’s modified Eagle’s medium high-glucose (DMEM; Sigma, St. Louis, MO, USA) supplemented with heat-inactivated 10% fetal bovine serum (FBS) (Gibco-BRL, Gaithersburg, MD, USA) and 2 mM L-glutamine. Briefly, cells were seeded in 96-well plates; subconfluent cell monolayers were treated with different volumes of theranostic nanodroplets (ranging from 0.39 to 50 µL per well). After 24 h of incubation, cell viability and cytotoxicity were assessed. Cell viability was determined using the CellTiter 96 Proliferation Assay Kit (Promega, Madison, WI, USA) according to the manufacturer’s instructions. Absorbance was measured using a microplate reader (Model 680, Bio-Rad Laboratories, Hercules, CA, USA) at 490 nm. The effect on cell viability was expressed as a percentage, by comparing the absorbance of the treated cells with that of those incubated with the culture medium alone. Cytotoxicity was determined by assessing the level of lactate dehydrogenase (LDH) released in the culture supernatants, by using the CytoTox 96 Non-Radioactive Cytotoxicity Assay Kit (Promega, Madison, WI, USA), following the manufacturer’s instructions. The cytotoxic effect of theranostic nanobubbles was expressed as a percentage, by comparing the absorbance of the treated and untreated samples with that of cells incubated with the lysis buffer provided by the kit.

### 2.9. Cellular Uptake Experiments

The mouse melanoma (B16-F10) cell line was purchased from the American Type Culture Corporation. Cells were cultured in Dulbecco’s modified Eagle’s medium (DMEM) containing 4.5 g/L glucose, 10% (*v*/*v*) FBS, 2 mM glutamine, 100 U/mL penicillin, and 100 μg/mL streptomycin, and then they were incubated at 37 °C in a humidified atmosphere of 5% CO_2_. At 80% confluency, the cells were detached with trypsin–EDTA solution, centrifuged, and dispensed into new flasks. 

The human prostate cancer (PC-3) cell line was purchased from the ATCC. Cells were cultured in DMEM-F12 supplemented with 2 mM glutamine, 10% FBS, and penicillin/streptomycin antibiotics (10,000 IU/mL penicillin, 10,000 IU/mL streptomycin), and then they were incubated at 37 °C in a humidified atmosphere of 5% CO_2_. At 80% confluency, the cells were detached with trypsin–EDTA solution, centrifuged, and dispensed into new flasks. 

For the uptake experiments, 5.0 × 10^4^ B16 or PC-3 cells were seeded in sterile tissue culture-treated ibidi open μ-Slides (chambered coverslips—dimensions of wells: w × l × h; in mm: 9.4 × 10.7 × 6.8) and treated with tissue culture (ibidi GmbH, Planegg, Germany). Then, 180 µL of DMEM or DMEM-F12 was added to each of the wells. After 24 h, the culture medium was discarded, and cells were incubated for 60 min with nanodroplets containing PLP and coumarin 6 (diluted 1:16.7 in DMEM), resulting in a PLP concentration of 120 μg/mL. For B16 cells, the control was represented by cells incubated with complete medium only, while for PC-3 cells the control was represented by cells incubated with nanobubbles without PLP or with culture medium only. At the end of incubation, cells were washed four times with PBS and fixed with 4% formalin at room temperature for 15 min. The cells were then profusely washed again; nuclear staining with DAPI was performed for 20 min, followed by 5 min of washing in PBS. Finally, the cells were imaged through a Leica TCS SP5 confocal microscope (Leica Microsystems S.r.l.) at 40× magnification (laser 1 wavelength = 405 nm, laser 2 wavelength = 488 nm, to visualize Hoechst and coumarin 6, respectively). The experiments were performed in triplicate (n = 3).

### 2.10. Relaxometric Measurements

Longitudinal water proton relaxation time (T1) values were measured on a Stelar Spinmaster spectrometer (Stelar S.n.c., Mede (PV), Italy) operating at 0.5 T (21.5 MHz proton Larmor frequency), by means of the standard inversion–recovery technique (16 experiments, 2 scans). A typical 90°pulse width was 3.5 s, and the reproducibility of the T1 data was ±0.5%.

^1^H-NMRD profiles were measured at 37 °C on a Stelar Spinmaster-FFC field-cycling relaxometer by measuring T_1_ at magnetic field strengths from 2.4 × 10^−4^ to 0.47 T (corresponding to proton Larmor frequencies from 0.01 to 20 MHz). This relaxometer works under complete computer control with an absolute uncertainty in 1/T_1_(=R_1_) of ±1%. Additional points, from 0.47 T (20 MHz) to 1.7 T (70 MHz), were collected on a manually tunable Stelar Spinmaster spectrometer working under a variable magnetic field.

The temperature was controlled by a Stelar VTC-91 airflow heater equipped with a copper–constantan thermocouple; the actual temperature in the probe head was measured with a Fluke 52 k/j digital thermometer (Fluke AG, Zürich, Switzerland), with an uncertainty of ±0.2 °C.

The amount of Gd internalized into the nanobubbles was determined by T1 measurement at 21.5 MHz and 25 °C, after sample mineralization in 6 N HCl at 120 °C.

### 2.11. Ultrasound Imaging 

US imaging was performed using a VEVO-2100 device (Fujifilm VisualSonics, Toronto, ON, Canada). In vitro experiments were carried out using a phantom constituted by a tank made of polyethylene tubes immersed in deionized water. US images were acquired at different temperatures (25 °C and 37 °C) and transducer frequencies (21 MHz, 40 MHz, and 50 MHz). The in vivo experiment was carried out on an athymic nude mouse bearing a PC-3 subcutaneous prostate tumor; 5 × 10^6^ PC-3 cells were subcutaneously injected into the right flank of the animal. Tumor development was determined by caliper measurement twice a week, from 7 days after induction. Tumor volume was calculated according to the formula (L × W2)/2, where L and W are the maximum length and width of the tumor, respectively. A volume of 180 µL of nanobubbles (corresponding to ca. 2.2 × 10^6^ NBs) was injected into the tail vein of the animal, bearing a 200 mm^3^ tumor. 

All of the procedures involving animals were conducted according to the national and international laws on experimental animals (L.D. 26/2014; Directive 2010/63/EU; authorization: 229/2016-PR). No validated non-animal alternatives are known to meet the objectives of the study.

Ultrasound images were acquired in linear contrast mode, using a 40 MHz transducer, and following the changes in B-Mode amplitude over a total time of 2 min. 

### 2.12. Statistical Analysis

The results are expressed as the mean ± SD. The sample size (n) is reported for each experiment.

## 3. Results

### 3.1. General NB Characterization 

Preliminary experiments were carried out to tune the drug payload in the nanoformulation by evaluating the solubility and partition coefficient of PLP between the perfluoropentane and aqueous phases.

The PLP partition coefficient values determined in perfluoropentane and the perfluoropentane–DSPC mixture were 2.1 and 1.3, respectively, confirming the good affinity of PLP for the NBs’ oily inner phase. However, due to the amphiphilic property of the drug, it will likely localize at the interface between the shell and the perfluoropentane core. The lower partition coefficient measured in the presence of the amphiphilic DSPC molecules, which act as a surfactant monolayer at the PFP interface, could be due to the decrease in the space available for the drug at the interface. 

Once the interface is saturated, it is expected that PLP will self-aggregate, forming micelles. 

The critical micellar concentration (CMC) is the concentration at which, and above which, a surfactant forms micellar aggregates. The CMC of PLP was determined by measuring the surface tension of the PLP solutions. A CMC value of 5 mg/mL (11.3 mM) was obtained.

These results support the view that the drug is located at the PFP interface, and because the chemical structure of Gd-DTPAMA-CHOL does not differ too much from that of PLP, we speculate that the MRI agent also shares the same location in the NBs. 

The loading capacity of PLP and Gd-DTPAMA-CHOL was quite good, with values of 7.8% and 1.8%, respectively, while the encapsulation efficiency of both compounds was greater than 90%.

A series of NBs were prepared and characterized (see Table 1). 

In addition to the two samples with and without the theranostic companions, two other systems were formulated, differing in the loading of two fluorescent dyes located in the PFP core (the hydrophobic coumarin 6) or in the core–shell interface (the amphiphilic rhodamine–DSPE).

The NBs’ sizes ranged from approximately 540 nm to 560 nm. The size dispersion was very good, with PDI values slightly higher than 0.1. Due to the coating of glycol chitosan, the zeta potential values were positive, from about 13.4 mV to 17.6 mV. Here, the most positive potential was measured for the blank formulation, whereas the remaining NBs displayed lower values owing to the presence of the negatively charged PLP and Gd-DTPAMA-Chol. The pH value of the NB suspension was 6.2, with a viscosity of 1.45 cP and an osmolarity of 383 mOsm—values that highlight the suitability of the formulation for in vivo applications.

**Figure 2:**
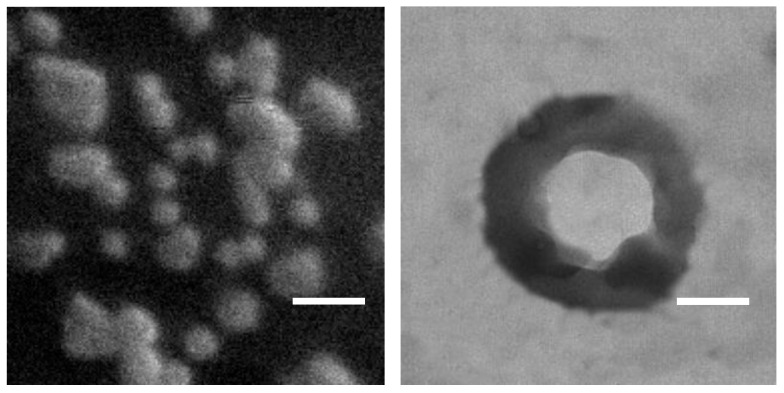
Electron microscopy of Gd-DTPAMA-Chol/PLP NBs. **Left**: SEM image, magnification = 10,000×, scale bar = 1 µm. **Right**: TEM image of a single particle, magnification = 52,000×, scale bar = 150 nm.

The NBs’ morphology was verified by SEM and TEM. NBs appeared as submicron spheres with a transparent perfluoropentane core and a thick polymeric shell (Figure 2).

The NBs’ physical stability was assessed by monitoring their size and Z-potential values over time for both blank and theranostic NBs. Considering the stability of the blank formulation, no changes in the physicochemical parameters were observed up to 3 months of storage at either 4 °C or 25 °C.

Table 2 reports the size, polydispersity index, and Z-potential values of the Gd-DTPAMA-Chol/PLP NB formulation over time, up to 3 months. Interestingly, at each timepoint evaluated, the average diameter and polydispersity index values did not increase, indicating the physical stability of the nanosystem. These results confirmed the role of the crosslinked polymeric hard shell.

Conversely, upon short US stimulation (20 s), the NBs’ size increased, following the vaporization of the PFP core (Table 3). 

**Figure 3:**
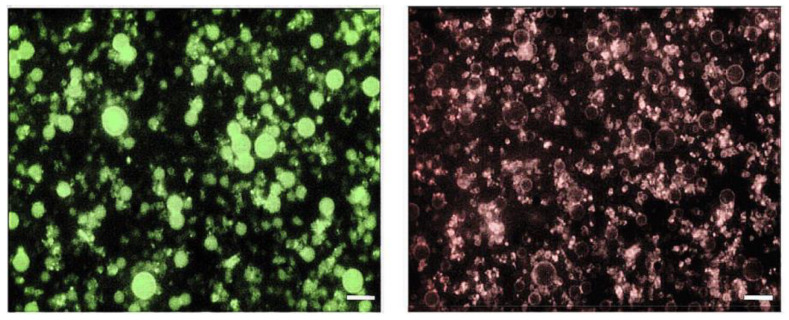
Fluorescence microscopy of theranostic (Gd-DTPAMA-Chol/PLP) NBs. The green signal (coumarin 6, **left**) corresponds to the perfluoropentane core, whereas the red signal (rhodamine–DSPE, **right**) corresponds to the polymeric shell (magnification = 630×, scale bar = 1 µm).

Fluorescence microscopy of NBs loaded with the lipophilic coumarin 6 or the amphiphilic rhodamine–DSPE dyes confirmed the expected locations of the fluorescent molecules, with coumarin 6 filling the PFP core and rhodamine–DSPE mainly localized at the PFP interface (Figure 3).

The size enlargement promoted by the short US stimulation, and likely due to the nanodroplets’/nanobubbles’ transformation, was clearly detected by looking at the fluorescent signal of rhodamine–DSPE, as displayed in Figure S1. 

Finally, the NBs’ physical stability was also evaluated in rat plasma at 37 °C after 3 h of incubation. During this time, the NBs were stable and maintained their morphology without undergoing aggregation. Moreover, the theranostic nanoformulation did not show hemolytic activity.

### 3.2. In Vitro Release Kinetics of PLP and Gd-DTPAMA-CHOL from Nanobubbles

**Figure 4:**
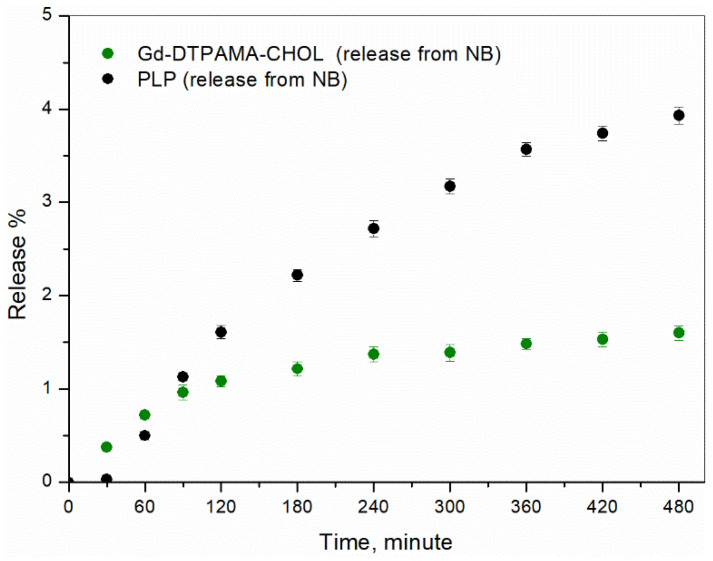
Release kinetics at 37 °C (in buffer) of PLP (black circles) and Gd-DTPAMA-CHOL (green circles) from the theranostic nanobubbles (n = 3).

The in vitro release kinetics of PLP and Gd-DTPAMA-CHOL were evaluated at 37 °C and in the presence and absence of US stimulation. A slow and prolonged release of the two companions was observed. This behavior confirms the strong incorporation of PLP and Gd-DTPAMA-CHOL in the nanostructure (Figure 4). 

Both of the compounds displayed a pseudo-hyperbolic profile with a release lower than 4% after 12 h in the absence of US. However, Gd-DTPAMA-CHOL exhibited a slower release, starting from 2 h. In fact, whereas the long-term release of PLP reached a cumulative value of 4% (after 8 h), the amount of released Gd(III) complex at the same time was ca. 1.5%. 

We analyzed the release profiles with different kinetic models (zero-order, first-order, Higuchi, and Korsmeyer–Peppas), and the Higuchi model showed by far the best performance, with the highest square correlation coefficient (R^2^) value (0.97) for both compounds. This result indicates that the release kinetics is controlled by diffusion through the polymer shell.

**Figure 5:**
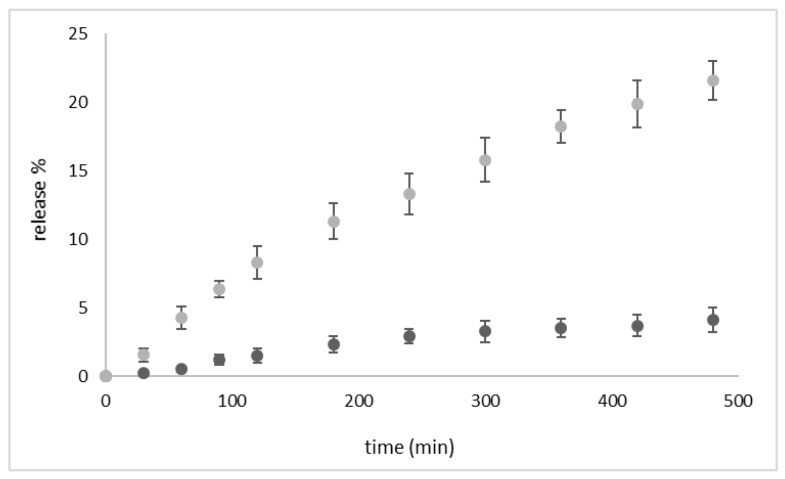
In vitro release kinetics of PLP from the theranostic nanobubbles at 37 °C (in buffer) in the absence of US (dark-grey-filled circles) and upon US exposure (grey-filled circles, US frequency 2.5 ± 0.1 MHz, 5 min of insonation) (n = 3).

Figure 5 shows that upon a single application of US the in vitro release of PLP significantly increased, with an enhancement of ca. 450% after 8 h.

This result is consistent with our previous work on theranostic chitosan nanobubbles, where the effect of US stimulation on PLP release enhanced the drug release [22].

### 3.3. Relaxometric Characterization

**Figure 6:**
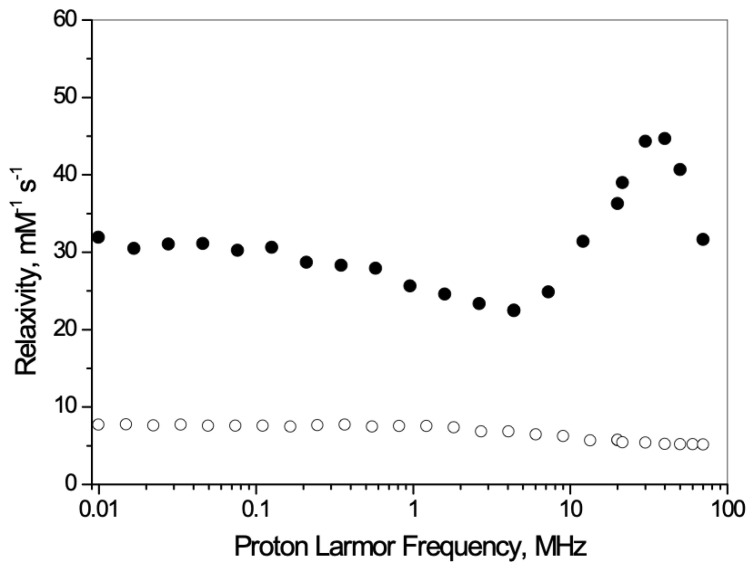
NMRD profiles recorded at 37 °C for Gd-DTPAMA-CHOL: free (○) and loaded in the NBs (●).

Figure 6 reports the proton nuclear magnetic relaxation dispersion (NMRD) profiles recorded at 37 °C for the paramagnetic complex Gd-DTPAMA-CHOL, both free and incorporated in the NBs. 

The data indicate that when the Gd(III) complex was fully loaded in the nanobubbles, the relaxivity values were much higher than those of the free molecule over the entire range of magnetic field strengths investigated.

Furthermore, the shape of the profile of the complex embedded in the nanobubbles is typical for a paramagnetic complex with restricted rotational motion, which is invariantly characterized by a hump/peak whose maximum generally falls in the 10–40 MHz range [33].

**Figure 7:**
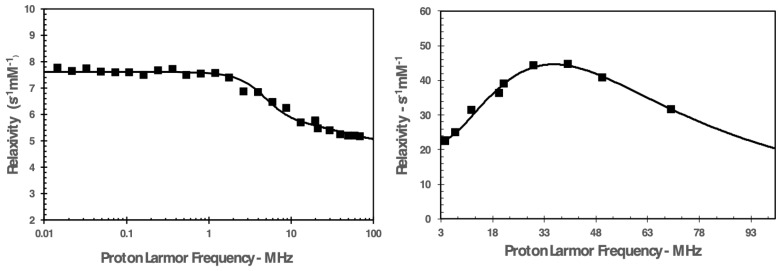
(**Left**): NMRD profile (at 37 °C) of Gd-DTPAMA-CHOL analyzed according to the SBM and Freed relaxation models. (**Right**): High-field portion of the NMRD profile (37 °C) for the nanobubbles loaded with Gd-DTPAMA-CHOL. Data were analyzed only in the reported magnetic field interval, according to the SBM and Freed relaxation models.

The NMRD profile of the free complex was analyzed according to the paramagnetic relaxation model based on the Solomon–Bloembergen–Morgan (SBM) and Freed theories (Figure 7, left) [34]. Table 4 reports the values of the more relevant parameters obtained from the data fitting.

The analysis did not allow for the determination of an accurate value for the residence lifetime of the water protons coordinated to the Gd(III) ion (t_M_), but the quality of the data interpolation was sufficiently good in the t_M_ interval 300–500 ns. A residence lifetime in this range is in agreement with data from the literature obtained for DTPA monoamide complexes [35,36], and the same applies for both the rotational correlation time of the complex (t_R_) and the parameters describing the relaxation of the unpaired electrons of the paramagnetic ion (D^2^ and t_V_).

As electronic relaxation parameters have no real physical meaning for nanosystems, the data acquired for the paramagnetic nanobubbles at magnetic fields lower than 3 MHz were not included in the analysis (Figure 7, right) [37]. The rotational tumbling of the complex embedded in the NBs was significantly reduced. On the other hand, the shortening of the residence lifetime of the water molecule bound to the Gd(III) ion could be attributable to the catalysis of the prototropic exchange of the metal-bound water protons occurring at the hydrophilic NB shell [37].

### 3.4. Cell Uptake and Viability

Confocal fluorescence microscopy demonstrated a very rapid, massive, and homogeneous uptake of coumarin-6-labeled nanobubbles. The green signal was appreciable in the cytoplasm of B16 and PC-3 cells (Figure S2), whereas in control cells no signal was detected. 

To investigate the effects of theranostic NBs on human cells, cytotoxicity and cell viability assays were performed. The results are displayed in Figure S3 and show that the proliferation of both HeLa and Helf cells was impaired as a function of the amount of NBs used. The Helf cell line was more sensitive than HeLa cells to the treatment with nanobubbles. The results of the cytotoxicity assays showed a slight cellular lysis only for Helf cells. This effect might be related to the presence of PLP. Indeed, Cittadino et al. previously showed that PLP incorporated in liposomes remarkably inhibited tumor growth in mice [38]. Blank nanobubbles did not affect the cell viability on either cell line.

The same behavior was also observed in cancer cells, i.e., the PC-3 cell line (Figure S4). Indeed, the blank NBs showed no cytotoxic effect on PC-3 cells, confirming their biocompatibility. On the contrary, theranostic NBs influenced the PC-3 cells’ viability. In particular, a reduction in cell viability was observed at higher amounts of NBs.

### 3.5. US imaging Detection of the Theranostic Nanobubbles

**Figure 8:**
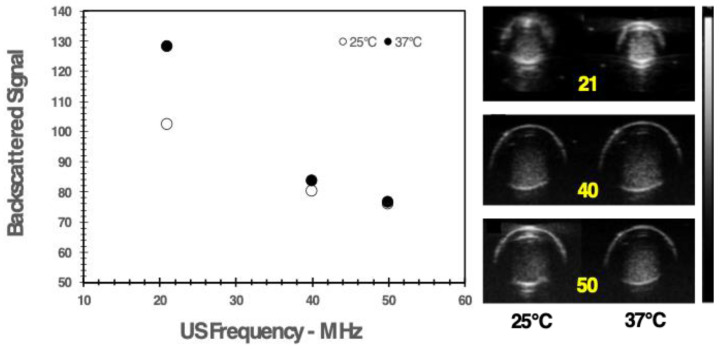
In vitro backscattered signal of the nanobubbles measured at different frequencies and temperatures. Right: axial view of the tubes containing the nanobubbles.

The NBs’ echogenicity was first tested in vitro using a customized phantom made of a series of polyethylene tubes. The NBs (1.2 × 10^7^ particles/mL) were heated to 37 °C, and B-Mode images of the phantom were acquired at different frequencies: 21 MHz, 40 MHz, and 50 MHz. The observed US signal showed a pseudo-exponential decay versus frequency (Figure 8). 

**Figure 9:**
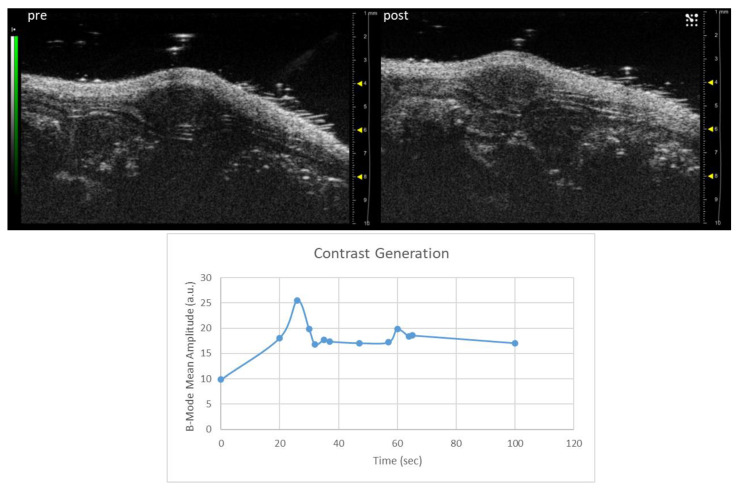
(**Top**) Representative image of a PC-3 xenograft tumor acquired in linear contrast mode before and 25 s after the injection of NBs. (**Bottom**) Changes in echo power (a.u.) observed in the tumor over time after the i.v. injection of the NBs.

The US detectability of the nanobubbles was also preliminarily tested in vivo. An athymic nude mouse bearing a xenograft prostate tumor was injected intravenously with a total volume of 180 µL of NBs (1.2 × 10^7^ NBs/mL). Linear contrast-enhanced US imaging was performed using the 40 MHz transducer. A spike in the signal was observed in the subcutaneous tumor after 25 s of injection (Figure 9), corresponding to the passage of the NBs.

### 3.6. Discussion

In this work, novel theranostic NBs for dual MRI/US imaging were developed, aimed at designing a precision nanomedicine tool. The rationale was to obtain a theranostic nanoplatform enabling the real-time visualization of the biodistribution of the intact system by US imaging, whereas the release of the drug (eventually triggered by ultrasound) would be monitored by MRI. Here, NBs were optimized for loading and stability—crucial points for the performance of the nanoplatform in future preclinical validation.

The formulation comprises a hard shell composed of glycol chitosan crosslinked with TPP and an inner core filled with perfluoropentane. Interestingly, the application of a short US stimulation may cause the vaporization of the PFP core and the transformation of the nanodroplets into nanobubbles, which displayed good stability at 37 °C. The effect of US to induce the phase transition of the perfluorocarbon core is known as acoustic droplet vaporization (ADV) [39]. Due to this liquid-to-gas transition phenomenon, the transformation of nanodroplets to nanobubbles may significantly improve their detection by US imaging [13,40,41].

In addition to facilitating imaging, ultrasound can also stimulate the release of the material transported by the nanocarrier, i.e., the drug and the MRI agent [42,43], as well as affecting cell uptake [29]. Much research has focused attention on the development of various ultrasound-sensitive nanodroplets, showing their potential in drug delivery and targeting, as well as in imaging [44,45,46]. Here, the theranostic companions are represented by PLP and the MRI agent Gd-DTPAMA-CHOL (Figure 1). In the proposed system, MRI should monitor drug release; for this reason, the agent was selected by virtue of its structural similarity (presence of a steroid-like moiety) to PLP.

The partition coefficient and CMC determination of PLP allowed for the estimation of its amphiphilicity in order to predict the location of the molecule in the NBs. A similar localization was expected for the structural analog Gd-DTPAMA-CHOL. However, the larger hydrophilic head in the latter compound may affect its behavior with respect to PLP.

The shell composition is a key factor in NB formulations, affecting their half-life and their in vitro and in vivo performance [46].

Chitosan and its derivatives are attractive components for nanoparticles, considering their high biocompatibility, biodegradability, and low immunogenicity [47,48,49]. Interestingly, glycol chitosan is soluble at physiological pH. Previously, we designed glycol-chitosan-shelled nanobubbles to deliver and release doxorubicin in anaplastic thyroid tumor cells and taxanes to castration-resistant prostate cancer cells [50,51,52].

Hydrophobically modified glycol chitosan conjugates have also been used to design various theranostic delivery systems containing imaging agents and therapeutics [53]. Here, to enhance its stability, glycol chitosan was crosslinked with TPP to produce hard-shelled nanodroplets/bubbles with increased stiffness. Moreover, the negatively charged DSPC monolayer at the PFP interface can electrostatically interact with the positively charged glycol chitosan forming the shell. Electron microscopy confirmed the NB core–shell structure, and further support was provided by the NB images with fluorescent dyes able to localize in the core and at the core–shell interface (Figure 3).

The NB size was in the submicron range and was unaffected by the presence of the theranostic companions. The zeta potential for theranostic NBs decreased compared to the blank nanosystem due to the glycol chitosan’s partial charge neutralization by the negatively charged hydrophilic moieties of the theranostic companions. The size, morphology, and surface charge are key parameters that affect the fate of the NBs after in vivo administration and their accumulation in tumor tissue. 

Although the size of the theranostic NBs developed here was about 500 nm, this value could be still suitable for passive tumor targeting, exploiting the enhanced permeability and retention (EPR) effect. Indeed, due to the leaky vasculature in the tumor tissue, nanoparticles of 10–780 nm in size can extravasate and accumulate at the tumor site [54,55,56].

However, the NBs’ sizes could be further tuned and optimized by varying the shell composition and the interfacial components of the nanostructure. The NB size modulation was previously investigated, showing the possibility of formulating NBs of about 100 nm in size [13].

The hard-shelled NBs displayed excellent stability at 25 °C without showing changes in size, polydispersity index, or zeta potential over three months, and the same result was obtained in rat blood at 37 °C after 3 h of incubation. When exposed to a short US pulse, the NBs’ size almost doubled. It is likely that this process reflects a nanodroplet/nanobubble switch associated with the liquid-to-gas transition of the PFP core. The vaporization of the PFP core was also clearly visualized by fluorescence microscopy (Figure S1). 

The in vitro release kinetics study indicated that the two companions shared a similar pseudo-hyperbolic profile to that of PLP, which displayed a maximum release at 8 h of ca. 4%, whereas the release of Gd-DTPAMA-CHOL under the same conditions was about 1.5%. This finding may be an indication that the Gd(III) complex is more tightly bound to the nanobubble interface, likely because of the presence of two negative charges in the linker that connects the complex to the steroidic moiety. Interestingly, the very slow release kinetics observed for both of the molecules is a positive result, because it may limit the drug release in the bloodstream, thereby reducing adverse side effects. In order to investigate the drug/MRI probe release mechanism, mathematical models were considered. The Higuchi model, which describes the diffusion-controlled release from a polymeric matrix, showed the best agreement with the experimental data for both compounds. This finding underlines the key role played by the polymeric shell and the stability of the crosslinked coating.

To induce the release of the components once the theranostic NBs reach the pathological site, the application of US is one of the most successful options. The application of a single dose of US to the theranostic nanobubbles significantly enhanced the release of the drug by ca. 450% after 8 h. 

The effects caused by the application of US can be divided into mechanical and thermal, depending on the characteristics of the acoustic waves (acoustic pressure, intensity, frequency, insonation mode, etc.). The two effects may interact differently with NBs, potentially affecting the release mechanism that can mainly occur via the detachment of the loaded molecule caused by structural modifications at the DSPC interface.

In addition to boosting the drug release, the external application of US could also be exploited to improve the cell internalization and tumor accumulation of the NBs.

One of the most peculiar features of the NBs developed in this work is represented by their ability to report the drug release in real time by MRI. The signal intensity produced by a T_1weighted_-MR image in the presence of a paramagnetic agent roughly correlates to the local concentration of the agent and to its longitudinal millimolar relaxivity (r_1_), which is an intrinsic property of each agent that reflects several dynamic and structural features of the molecule [37], and allows for the prediction of the in vivo efficiency of an MRI agent. The analysis of the magnetic field dependence of r_1_ (NMRD profile) is the best available methodology to obtain an estimate of the molecular parameters that contribute to the relaxivity. Furthermore, it allows for the identification of the magnetic field strength where the capability of the agent to generate the contrast is the highest, driving the choice of the MRI scanner to perform the imaging session.

The NMRD profiles acquired for Gd-DTPAMA-CHOL (free or incorporated in the NBs) show a large relaxivity difference between bound and unbound agents (Figure 6), indicating that the measurement of the MRI T_1_ contrast may be very sensitive to the release of the contrast agent. The greatest difference was observed at 30 MHz, where the relaxivity of the paramagnetic nanobubbles (44.3 s^−1^mM_Gd_^−1^) was ca. 720% higher than the corresponding value of the unbound agent (5.4 s^−1^mM_Gd_^−1^), but even at the clinical field of 60 MHz the difference was still very high (ca. 570%).

The profiles were analyzed using the conventional models for paramagnetic relaxation (the SBM and Freed models), and the values of the parameters obtained from the analysis (Table 4) confirmed that the most relevant effect caused by the loading of the complex to the nanosystem was the severe restriction of the rotational motion of the agent, t_R_, from 0.11 ns to 3.95 ns. 

Secondly, the residence lifetime of the water molecule coordinated to the Gd(III) center in the bound agent was slightly shortened when compared with the free molecule. This could be explained by catalysis of the prototropic exchange of the metal-coordinated water protons operated by the polar moieties of the NB hydrophilic shell. This hypothesis could be a further indication of the possible binding between the coordination cage of the Gd complex and the NB shell, which makes the loading of the MRI agent more stable with respect to PLP after 2 h. It is worth noting that the shortening of t_M_ for a paramagnetic complex with a restricted rotational motion can be very beneficial in terms of relaxivity. In fact, the r_1_ measured for the theranostic NBs was quite high for an agent with a single water molecule coordinated to the metal center. 

Some preliminary experiments were carried out to evaluate the interaction between cells and the theranostic nanosystem in terms of NB uptake, cell viability, and cytotoxicity. Melanoma B16 cells massively took up the NBs after 1 h of incubation. This result can be explained in terms of the electrostatic interaction between the positively charged NBs and the negatively charged cell membrane, which makes the cell internalization more efficient [57].

Interestingly, it is well known that cationic nanocarriers show strong cellular interaction properties and cellular uptake [58]. A similar behavior has already been observed in our previous studies, in which glycol chitosan NBs were rapidly internalized in cancer cells in a dose-dependent manner [21]. The mechanism of cell uptake of the theranostic NBs will be investigated in depth in the future. Blank NBs did not show any effect on cell viability and cytotoxicity. However, the presence of PLP caused a dose-dependent reduction in viability and an increase in cytotoxicity in the two cell lines examined, with Helf cells being more sensitive to the drug than HeLa cells. 

Finally, the NB echogenicity was tested in vitro and in vivo on a preclinical US imager. In vitro, the US signal generated by the sample was measured at different acoustic frequencies. The signal displayed a decrease as a function of frequency (Figure 8). The frequency dependence of the US signal may depend on many factors, such as the size and composition of the nanoparticles, and the mechanical index (MI) associated with the applied US waves. The latter is directly dependent on the acoustic pressure and inversely dependent on the square root of the frequency. Typically, echogenicity is proportional to MI [39], provided that MI is inferior to the limit at which the particles start to become unstable. As, in this experiment, the acoustic pressure was kept constant and the same sample was tested, the frequency dependence of the signal can be mostly ascribed to the frequency-induced effect on MI. 

Unfortunately, the 21 MHz transducer for in vivo imaging was unavailable; nevertheless, even using the 40 MHz transducer, a maximum signal was detected in a prostate cancer xenograft on a mouse 25 s after the NB intravenous injection. This is the proof of concept of the theranostic capability of this nanosystem.

## 4. Conclusions

In summary, a novel dual US/MRI nanoplatform for the real-time visualization of drug delivery and release was prepared and preliminarily characterized. The nanosystem consisted of perfluoropentane-cored NBs stabilized by a phospholipid layer whose stiffness was augmented by a crosslinked glycol chitosan shell and loaded with Gd-DTPAMA-CHOL and the prodrug PLP. The structural similarity of the compounds allows for their localization at the NB interface, guaranteeing similar release kinetics.

In principle, the NBs molecules can be visualized either by US or MRI, although the in vivo MRI detection was prevented by the limited amount of MRI loaded in the current formulation. Both imaging modalities may generate a signal dependent on the amount of intact nanobubbles, but in vitro experiments suggested that MRI contrast can be also modulated (reduced) by the release of the paramagnetic complex. Therefore, a comparison between the temporal evolution of the two imaging responses could qualitatively report on the occurrence of the release.

This work represents an initial proof of concept that provides a glimpse of the good potential of this approach for developing an image-guided drug delivery protocol that is able to monitor real-time drug release for improving personalized therapies.

## Figures and Tables

**Table 1:** Physicochemical characteristics of NB formulations.

Hard-Shelled Nanodroplets Formulation	Average Diameter ± SD (nm)	Polydispersity Index	Z-Potential ± SD (mV)
Blank nanodroplets	538.7 ± 24.7	0.13	+17.6 ± 2.9
Gd-DTPAMA-Chol/PLP	540.2 ± 30.5	0.11	+14.3 ± 2.5
Gd-DTPAMA-Chol/PLP/coumarin 6	562.6 ± 41.4	0.11	+14.1 ± 1.4
Gd-DTPAMA-Chol/PLP/rhodamine–DSPE	553.3 ± 38.2	0.11	+13.4 ± 1.2

**Table 2:** Physical stability of theranostic NBs over time at 25 °C. Size, polydispersity index, and Z-potential values determined just after preparation, and after 24 h, 96 h, and 15, 30, and 90 days.

	Average Diameter ± SD (nm)	Polydispersity Index	Z-Potential ± SD (mV)
Time 0	545.6 ± 18.4	0.110	+12.9 ± 3.2
24 h	550.1 ± 19.5	0.112	+13.4 ± 4.0
96 h	551.3 ± 13.6	0.111	+13.2 ± 3.1
15 days	547.6 ± 15.2	0.112	+12.8 ± 4.3
30 days	550.8 ± 12.5	0.108	+13.0 ± 3.5
90 days	548.4 ± 17.3	0.109	+13.5 ± 2.5

**Table 3:** Size, polydispersity index, and Z-potential values for the theranostic (Gd-DTPAMA-Chol/PLP formulation) NBs before and after the short US application.

	Average Diameter ± SD (nm)	Polydispersity Index	Z-Potential ± SD (mV)
No US	534.3 ± 12.6	0.111	+13.1 ± 4.5
Short US application	1088 ± 22.8	0.125	+13.3 ± 2.4

**Table 4:** Relaxation parameters (at 37 °C) obtained from the analysis of the NMRD profiles ^a^.

	Δ^2^ × 10^19^ (s^−2^)	^310^t_V_ (ps)	^310^t_R_ (ns)	^310^t_M_ (ns)
Gd-DTPAMA-CHOL	4.8	24.5	0.11	300–500
Theranostic nanobubbles	1.2	11.1	3.95	150

^a^ The distance of the coordinated water molecule from the metal ion (r^Gd–H^ = 3.02 Å) and the distance of the closest approach for the outer-sphere water protons (a = 3.7 Å) were determined from the analysis of the NMRD profiles of free Gd-DTPAMA-CHOL and were kept fixed in the analysis of the nanobubbles. A relative diffusion coefficient of solute and solvent (D) of 2.95 × 10^−5^ cm^2^s^−1^ was used.

## Data Availability

Data is contained within the article or Supplementary Material.

## Supplementary Materials

The following supporting information can be downloaded at: https://www.mdpi.com/article/10.3390/nano13152227/s1, Figure S1: Fluorescence microscopy images of NBs; Figure S2: confocal images of NBs uptake in cells; Figure S3: cell viability assay, Figure S4: Cytotoxicity assays. Reference [37] is cited in the supplementary materials.
