# Supplementary material for: Hard-Shelled Glycol Chitosan Nanoparticles for Dual MRI/US Detection of Drug Delivery/Release: A Proof-of-Concept Study
Source: Nanomaterials (Basel). 2023 Aug 1;13(15):2227. doi: 10.3390/nano13152227 (PMC10420971; doi:10.3390/nano13152227)
Supplement: Supplementary file 1 [file nanomaterials-13-02227-s001.zip › nanomaterials-2493554-Supplementary .pdf]

Supplementary Materials

Hard-Shelled Glycol Chitosan Nanoparticles for Dual MRI/US Detection of Drug Delivery/Release: A Proof-of-Concept Study

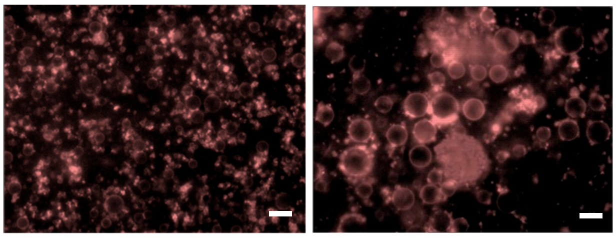

Figure S1. Fluorescence microscopy images of theranostic nanobubbles. The signal arose from rhodamine-DSPE. Left: 25 °C, right: at 37 °C after short US stimulation. Magnification 630X, scale bar 1μm.

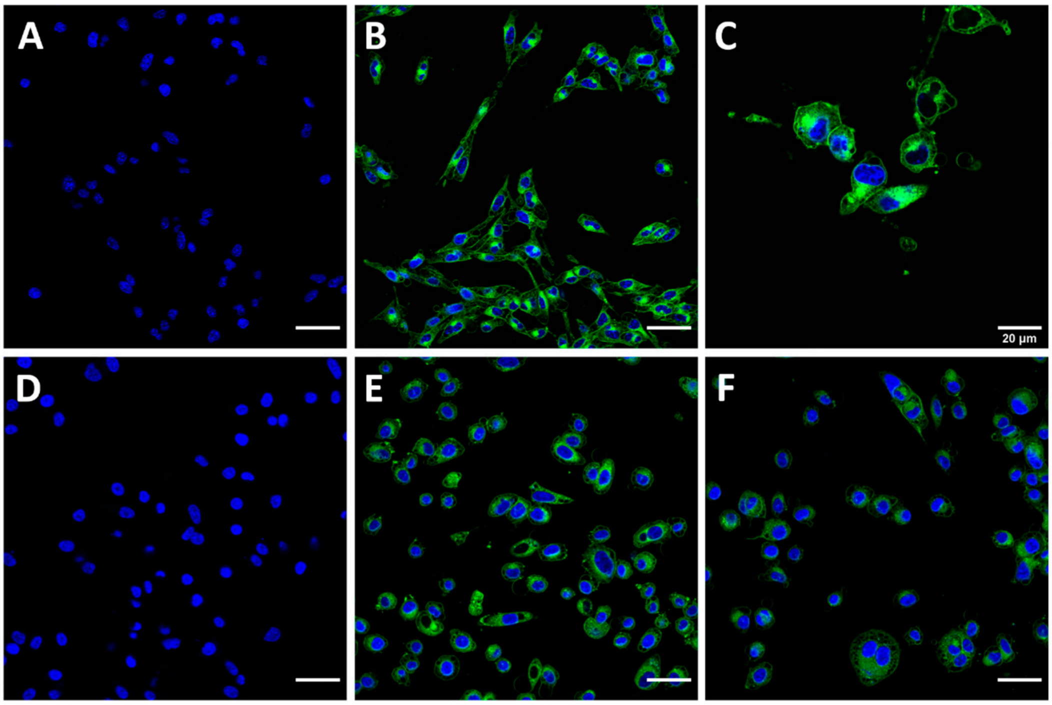

Figure S2. Confocal images (40x) of nanobubbles uptake in B16 and PC-3 cells: A) Control B16 cells, B) B16 cells incubated with fluorescent PLP-NBs, C) zoomed B16 cells incubated with fluorescent PLP-NBs, D) Control PC-3 cells, E) PC-3 cells incubated with fluorescent PLP-NBs, F) PC-3 cells incubated with fluorescent NBs. Nuclei were stained in blue, Coumarin 6 signal is represented in green. The scale bar corresponds to 50 μm unless otherwise stated.

In order to investigate the effect of theranostic nanobubbles on human cells, cytotoxicity, and cell viability assays were performed. The results displayed in Figure S3 show that the proliferation of both HeLa and Helf cells is impaired as a function of the amount of theranostic nanobubbles used for the treatment (panels A and C). Helf cell line resulted more sensitive than HeLa to the treatment with nanobubbles. The results of cytotoxicity assays (panels B and D) show a slight rate of cellular lysis only for Helf cells. This effect might be related to the presence of PLP. Indeed, Cittadino *et al.* previously shown that PLP incorporated in liposomes remarkably inhibited the tumor growth in mice.[1]

Blank nanobubbles did not affect the cell viability on both cell lines (data not shown).

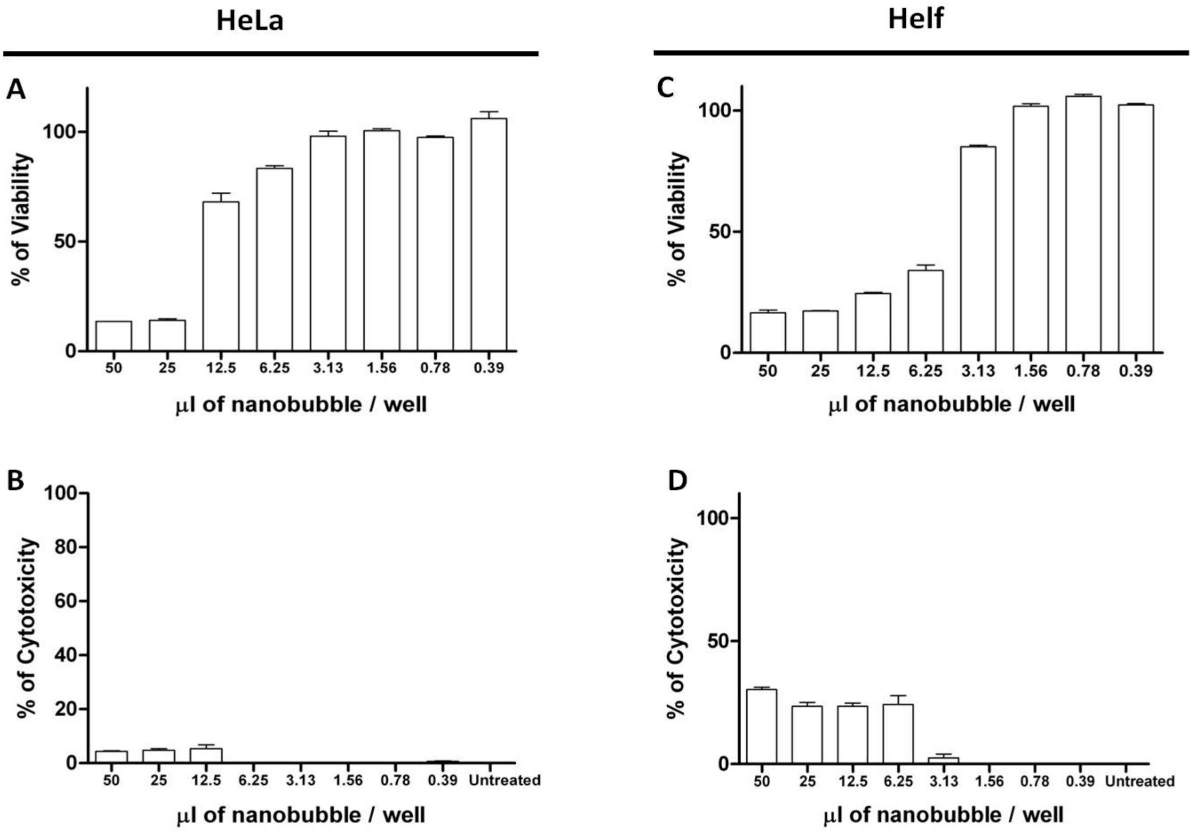

Figure S3. Effect of theranostic nanobubble treatment on human cell lines. Left: results of cell viability assays (A) and cytotoxicity assays (B) on HeLa cells. Right: results of cell viability assays (C) and cytotoxicity assays (D) on Helf cells (n=3).

The same behavior was observed also on cancer cells, i.e PC-3 cell line (Figure S4). Indeed, the blank NBs showed no cytotoxic effect on PC-3 cells confirming their biocompatibility. On the contrary theranostic NBs influenced the PC-3 viability. In particular, a reduction of cell viability was observed at higher amounts of NBs.

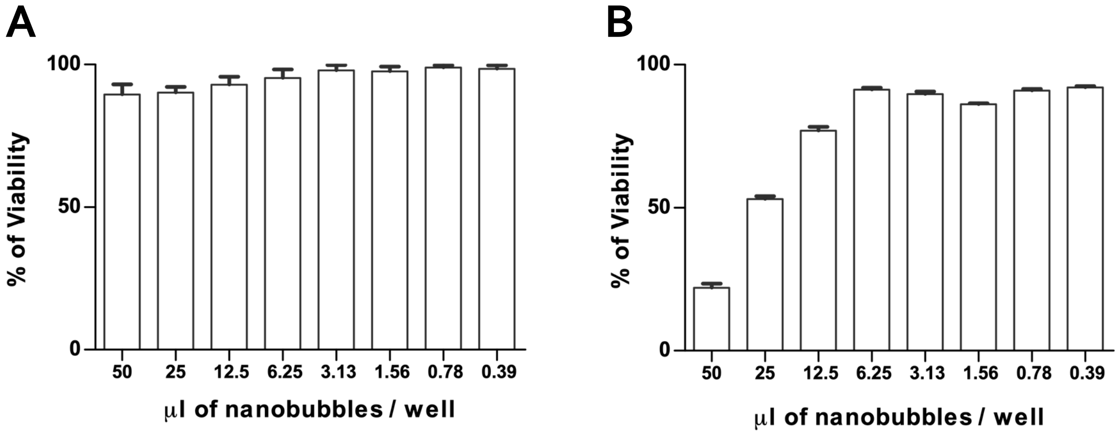

Figure S4. Cytotoxic effect of blank (A) and theranostic nanobubbles (B) on PC-3 cells (n=3).

References

1. Cittadino, E, Ferraretto, M, Torres, E, Maiocchi, A, Crielaard, BJ, Lammers, T, Storm, G, Aime, S, Terreno, E. MRI evaluation of the antitumor activity of paramagnetic liposomes loaded with prednisolone phosphate. 2012, Eur. J. Pharma. Sci. 45:436-441.
